# An anti-cancer Smurf

**DOI:** 10.1186/2045-3701-2-10

**Published:** 2012-03-19

**Authors:** Keji Zhao, Yun-Bo Shi

**Affiliations:** 1 Systems Biology Center, National Heart, Lung, and Blood Institute (NHLBI), National Institutes of Health (NIH), Bethesda, Maryland 20892, USA; 2 Section on Molecular Morphogenesis, Program in Cellular Regulation and Metabolism (PCRM), Eunice Kennedy Shriver National Institute of Child Health and Human Development (NICHD), National Institutes of Health (NIH), Bethesda, Maryland 20892, USA

## Abstract

A novel, cancer-fighting function was recently discovered for Smad ubiquitination regulatory factor 2 (Smurf2).

## 

A new guardian of genomic stability against cancerous mutations has been discovered, or so reported in a recent article published in Nature Medicine from Dr. Ying E. Zhang's laboratory at the National Cancer Institute in Bethesda, Maryland [1]. This new cancer fighter, Smurf2, was originally identified as Smad ubiquitination regulatory factor 2, which is an E3 ubiquitin ligase involved in the signaling control of the TGF-β and BMP super-family of poly-peptide growth factors [2].

Smurf2 and its related protein Smurf1 are members of HECT domain containing ubiquitin E3 ligases. Both of them play pleiotropic roles in many aspects of cellular functions ranging from regulating planar cell polarity during embryonic development to osteogenic differentiation in adult bone formation [3,4]. However, there had been little evidence supporting a connection of Smurfs to tumorigenesis prior to this work except for some sporadic reports of dysregulation of Smurf2 expression in breast and esophageal cancers and that up-regulation of Smurf2 induces senescence, which suppresses tumor cell proliferation [5]. The latest study from the Zhang group reported that mice deficient in Smurf2 are prone to various types of cancers at old age despite being able to develop normally. To investigate the underlying mechanisms of increased cancer development, the Zhang group established immortalized lines of embryonic fibroblasts from Smurf2-deficient embryos and found that these cells have altered patterns of histone modifications and loosely compacted chromatin. As a result of such epigenetic changes, the genomes of these cells become unstable, and the cells have already gone through the oncogenic transformation. Since the epigenetic alterations also occur in the spleen and primary dermal fibroblasts freshly isolated from Smurf2-deficient mice, loss of Smurf2 likely initiates a cascade of events early on in the life of affected mice that culminate in the tumor formation as they age. Indeed, the Zhang group identified RNF20, which itself is an E3 ligase for the monoubiquitination of histone H2B, as a new substrate of the E3 ubiquitin ligase activity of Smurf2. They further showed an inverse relationship between Smurf2 and RNF20 expression in human breast cancer tissues, corroborating the functional interplay of these two enzymes in humans. The significance of the findings by Blank et al. [1] lies in the fact that they established a direct link between specific changes in chromatin modification and cancer and provided a useful mouse model of cancer-causing epigenetic lesions for future mechanistic studies and drug testing.

## Competing interests

The authors declare that they have no competing interests.

## Authors' contributions

KZ and YBS wrote the manuscript. All authors read and approved the final manuscript.
